# Nanoporous Amorphous Carbon with Exceptional Ultra-High Strength

**DOI:** 10.3390/nano13081429

**Published:** 2023-04-21

**Authors:** Daniel Castillo-Castro, Felipe Correa, Emiliano Aparicio, Nicolás Amigo, Alejandro Prada, Juan Figueroa, Rafael I. González, Eduardo Bringa, Felipe J. Valencia

**Affiliations:** 1 Centro de Nanotecnología Aplicada, Facultad de Ciencias, Universidad Mayor, Santiago 7500994, Chile; 2 Escuela de Ingeniería en Computación e Informatica, Facultad de Ciencias, Universidad Mayor, Santiago 7500994, Chile; felipe.correa@mayor.cl; 3 CONICET and Universidad de Mendoza, Mendoza 5500, Argentina; 4 Facultad de Ingeniería, Arquitectura y Diseño, Universidad San Sebastián, Bellavista 7, Santiago 8420524, Chile; 5 Departamento de Computación e Industrias, Facultad de Ciencias de la Ingeniería, Universidad Católica del Maule, Talca 3480112, Chile; 6 Centro Para el Desarrollo de la Nanociencia y Nanotectonolgía, CEDENNA, Estación Centtral 917022, Chile

**Keywords:** amorphous carbon, molecular dynamics, plasticity

## Abstract

Nanoporous materials show a promising combination of mechanical properties in terms of their relative density; while there are numerous studies based on metallic nanoporous materials, here we focus on amorphous carbon with a bicontinuous nanoporous structure as an alternative to control the mechanical properties for the function of filament composition.Using atomistic simulations, we study the mechanical response of nanoporous amorphous carbon with 50% porosity, with sp${}^{3}$ content ranging from 10% to 50%. Our results show an unusually high strength between 10 and 20 GPa as a function of the $\%sp^{3}$ content. We present an analytical analysis derived from the Gibson–Ashby model for porous solids, and from the He and Thorpe theory for covalent solids to describe Young’s modulus and yield strength scaling laws extremely well, revealing also that the high strength is mainly due to the presence of sp${}^{3}$ bonding. Alternatively, we also find two distinct fracture modes: for low $\%sp^{3}$ samples, we observe a ductile-type behavior, while high $\%sp^{3}$ leads to brittle-type behavior due to high high shear strain clusters driving the carbon bond breaking that finally promotes the filament fracture. All in all, nanoporous amorphous carbon with bicontinuous structure is presented as a lightweight material with a tunable elasto-plastic response in terms of porosity and sp${}^{3}$ bonding, resulting in a material with a broad range of possible combinations of mechanical properties.

## 1. Introduction

Nanoporous carbon materials have been typically referred to as one of the most lightweight materials synthesized to date. Taking advantage of the strength of their bonds, it is possible to achieve ultrathin filaments within a porous structure with large dimensional stability [1]. This feature has motivated the synthesis of nanostructures with the unprecedented surface to volume ratios [1,2,3,4], and relative densities close to the air. All these features have endowed nanoporous carbon with extreme properties such as ultra-low thermal conductivity, as well as turned them into energy devices with an overwhelming energy storage capacity [5].

The exceptional ability of carbon to form multiple allotropes has fueled the synthesis of several nanoporous carbons with novel filament structures, ranging from graphitic mostly conformed by filaments of sp${}^{2}$-coordinated atoms [6,7], to diamond-like nanofoams with a large population of struts with sp${}^{3}$-coordinated atoms [8,9,10]. More precisely, sp${}^{2}$ nanoporous carbons have been synthesized based on graphene-like structures, in both open and closed pore structures, or with graphitic-like nanostructures with low relative densities [11]. Furthermore, to date, the degree of control in the design of nanoporous carbons has been extremely refined for constructing nanolattices of pyrolytic carbons with excellent mechanical properties [12,13]. Other studies have used the mechanical properties of nanoporous carbons as a route for efficient gas liberation [14]. Interestingly, high content sp${}^{3}$ carbon foams have been produced on macro- and microscales using pristine and polycrystalline diamond filaments [15,16]. This achievement has motivated their use in supercapacitors [17] and thermal devices [18,19]. In addition, diamond-like nanofoams have been synthesized by hydrothermal carbonization [20] of sucrose and from repeated laser ablation of carbon materials [21,22], producing filament structures with a mixture of sp${}^{3}$ ranging from 20 to 45%. Both types of foams could be considered a unique group to perform studies of carbon foams under pressure.

Different from graphite or diamond materials, diamond-like carbons allow describing the full range of mechanical and thermal properties characteristic from low to high content of $\%sp^{3}$ [19,23,24,25], to the point that there are numerous studies to refine their synthesis methods [26,27]. Unfortunately, the lack of ductility of amorphous carbon materials has been one of their main shortcomings that limits their performance compared with other structural materials. In this aspect, some interesting contributions have improved carbon ductility in nanoparticles such as nanoporous design or hollow cavities [28,29]. The same approach has been used in other amorphous materials, such as CuZr metallic glasses, where a nanoporous structure has been used to delay the apparition of shear bands [30,31]. Mixing the outstanding strength of amorphous carbon materials in a porous design could take advantage of the characteristic strength of diamond-like materials, but in a lightweight and ductile structure. This is precisely the focus of this contribution.

Atomistic simulations have been raised as one of the most relevant tools in searching for novel carbon materials [32]. There has been a large effort to model graphene foams [33], hybrid graphene–fullerene foams [34], and graphene–CNT foams [35]. Mechanical properties of random graphene foams of different densities have been studied [36,37]. However, the simulation of mechanical properties under tension often suffers from problems due to inappropriate bond-breaking descriptions of the REBO and AIREBO potentials. A previous work delved into this matter by testing the effect of the so-called switching function, as well as comparing different potentials [38]. In this aspect, the environmental dependent interatomic potential [39] (EDIP) was shown to accurately describe the mechanical properties of amorphous carbon materials [19,24].

In this work, we use atomistic molecular dynamics simulations to study the mechanical properties of random amorphous carbon foams under tension, where density changes are associated with different hybridization content. Elasticity, plasticity, and failure modes were correlated with the $sp^{3}$ content.

## 2. Methods

Tensile deformation of nanoporous carbon was addressed by atomistic simulations using the LAMMPS code [40]. The interaction of carbon atoms was modeled using the environmental dependent interatomic potential (EDIP) [39]. There are many potentials available in the literature to simulate carbon materials [39,41,42,43]. However, only a few of them are capable of reproducing amorphous carbon (aC) in closeness to experimental results for a wide range of hybridization values [44,45]. EDIP can simulate the aC density with the corresponding %sp${}^{3}$ content in agreement with experimental evidence and ab initio calculations [46,47]. Even more, the mechanical properties such as Young’s modulus and hardness agree with experimental measures probed with nanoindentation techniques [24].

Before the construction of the nanoporous materials, we created a set of aC bulk specimens with different densities and sp${}^{3}$ ratios, but with the same simulation cubic box length *L*. This was performed using the protocol proposed by Marks et al. [46], which consists of quenching the molted carbon with a given density from a temperature of 5000 K to a target temperature of 300 K, using an exponential cooling rate as can be found in reference [46]. After each one of the aC bulk samples was constructed, the residual stress was eliminated by relaxing the sample at 0.2 ns at 300 K with a zero pressure barostat. The density of the initial molten carbon directly influences the final sp${}^{3}$ content. By controlling the density of the sample, we obtained aC bulks with %sp${}^{3}$ = 12.1, 14, 16, 18.2, 23.0, 35.1, 42.2, and 49.7%. All of the aC bulk samples have the same simulation cell length L=16.3 nm. We note that hybridization in classical simulations is calculated from the coordination number obtained from the pair correlation function up to a cutoff radius of 0.2 nm. Coordination from Voronoi polyhedra gives similar results.

**Figure 1:**
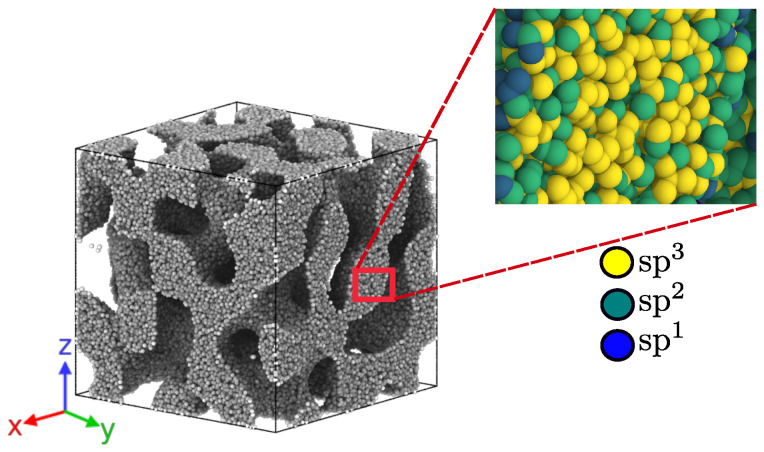
Nanofoam with a porosity of 50% and an sp${}^{3}$ content of 49.1%. Inset shows the hybridization of the C atoms on the foam filaments.

To construct the nanoporous structure, we adopted the algorithm proposed by Soyarslan et al. [48]. In their formalism, Soyarslan and co-workers assumed that a solution, $f(\vec{r})$, of the spinodal decomposition of a bicontinuous alloy is the sum of periodic waves with different wave vectors (${\vec{q}}_{i}$). The wave vector is defined as ${\vec{q}}_{i}=\frac{2\pi }{L}\left(h,k,l\right)$, with *L* the size of the simulation box (L=16.3 nm), and *h*, *k*, and *l* are integers termed as the Miller index of the sample. To choose the number of wave vectors and their respective directions, we follow the rule

(1) $$H=\sqrt{h^{2}+k^{2}+l^{2}}\;,$$

which satisfies that $\left|\vec{q}\right|=2\pi H/L$. To construct the nanoporous foam we choose H=14, which renders 24 wave vectors ${\vec{q}}_{i}$, satisfying condition (1). As the spinodal structure grows from the sum of periodic waves, it is expected that adding 24 waves to $f(\vec{r})$ gives a more representative structure than a solution constructed with just a few vectors. The solution of the spinodal decomposition applied over an amorphous carbon bulk follows the rule

$$f(\vec{r})\leq \epsilon \;,$$

where setting ϵ=0 defines a nanoporous material with a porosity close to 0.5. To understand the role displayed by the filament structure, we applied the same solution $f(\vec{r})$ over each aC bulk to obtain the same filament structure but with different sp${}^{3}$ compositions. An illustration of the resulting nanofoam with a given sp${}^{3}$ composition is illustrated in Figure 1. The sample size in our simulations are similar in size to the ones used in simulations of nanofoams including porous graphitic samples [36]. The filament size in experimental bicontinuous foams can reach nm sizes, as in our simulations. Bicontinuous foams with filaments smaller than a critical size, around 1.5 nm for Au foams, could display necking without any external load due to capillary forces [49]. We did not observe any such instability in our simulations. In order to study mechanical properties, the cross-section of the sample has to include at least a few filaments, in order to behave as a representative volume element inside a larger experimental sample, and this is the case for the simulated samples. In the presentation of the method used to build the samples in this study, it was shown that samples with porosity of 50% required a box side with about 7 filament diameters to obtain reliable elastic properties [48], and this is the case for our samples.

Cutting the nanofoams from each aC bulk sample previously constructed slightly reduced the average coordination of the samples because of the lower coordination of carbon atoms at the surface of the nanopores. Each aC nanofoam was relaxed again at 300 K with a zero pressure barostat for another 0.2 ns. The sample was simulated using an NVE ensemble, and the temperature was controlled using a velocity re-scale algorithm, with a timestep of 1.0 fs. The resulting nanofoams maintained a porosity of 50% with a %sp${}^{3}$ contents of 10.8, 13.9, 15.4, 17.9, 22.3, 34.7 41.8, and 49.1%. The obtained densities for the foams are listed in Table 1.

The mechanical properties of each specimen were calculated using purely uniaxial loading, keeping the sample cross-section constant to emulate high strain-rate deformation experiments. To this end, we assume fixed lateral periodic boundary conditions to mimic conditions under shock loading conditions. In our simulations, a strain rate of 1.0 $\times \;10^{8}$ s${}^{-1}$ was used. Even though the strain rates are faster than the observed in experiments, there are several theoretical works that show that strain rates of $1.0\;\times \;10^{8}$ s${}^{-1}$ are enough to capture elastic and plastic response of nanoporous materials [50]. Furthermore, Aparicio et al. [51] showed that there are not significant strain rate effects on graphene nanoribbons deformed at rates of 1.0 $\times \;10^{7}$ s${}^{-1}$ and 1.0 $\times \;10^{9}$ s${}^{-1}$, and Pedrielli et al. [36] used 1 $\times \;10^{9}$ s${}^{-1}$ for graphitic nanofoam deformation simulations.

During the whole deformation, the temperature was set constant at 300 K by means of a velocity rescale algorithm and a timestep of 1.0 fs.

System visualization and postprocessing were carried out with the OVITO code [52]. Carbon hybridization was obtained from the pair coordination function adopting a cutoff of 2.0 Å [44,46].

The software FoamExplorer [53] was used to study the diameter distribution properties of ligaments and voids of the samples. As amorphous structures were simulated, parameters were hand-picked based on the thickness of the resulting desired surfaces (composed of “external atoms” in the software’s terms), for each sp${}^{3}$ density case. Every 1% strain, ligament, and void diameter distribution were calculated for each sp${}^{3}$ density case. In some cases, normalization could be needed, which is a division of each diameter by the corresponding one at 0% strain. This requirement is caused by the irregularities of the surface for different sp${}^{3}$ concentrations and surface parameters for FoamExplorer.

## 3. Results

**Figure 2:**
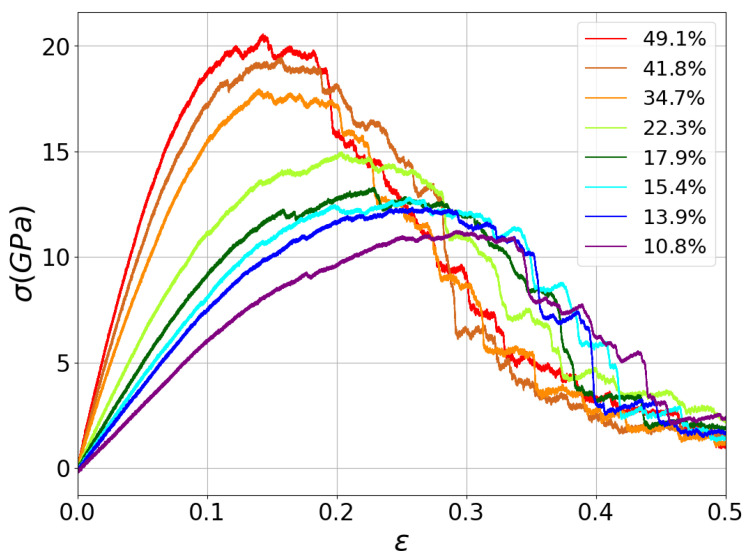
Stress–strain curve of the simulated nanofoams with different sp${}^{3}$ compositions.

Characterization of mechanical properties of aC nanofoams was derived from the stress–strain curves presented in Figure 2. For all the curves, the mechanical behavior of nanoporous carbon is dominated by the %sp${}^{3}$ content. These results are on the scale of experimental findings for different carbon foams [11,54,55]. It is straightforward to note the role displayed by the sp${}^{3}$ content, where larger diamond bonding is observed as Gaussian-like curves that drop abruptly after the ultimate tensile stress. In contrast, high-sp${}^{2}$ samples show a decrease in ultimate tensile stress, but with a prolonged plastic flow during the tensile test. In this way, the nanofoam inherits, in part, the phenomenology of the diamond-like films in terms of the sp${}^{3}$ bonding. Figure S1 show the Von Mises strain for sp${}^{3}$ = 49.1% and sp${}^{3}$ = 22.3%, which show that larger sp${}^{3}$ concentrations promote the stress localization, driving the filament breaking under some scenarios.

The material’s maximum tensile strength varies from 12.5 GPa for samples with $\%sp^{3}$ = 10% to ≈19 GPa for foams with $\%sp^{3}$ close to 50%. These values are smaller than those expected for aC bulk, which can reach a maximum tensile strength close to 60–80 GPa for diamond-like carbon films with high sp${}^{3}$ content. However, these values are surprisingly high compared with other nanoporous materials. For instance, a maximum tensile stress of 0.3 MPa is shown for nanoporous Au with porosities of 50%, 6 GPa for nanoporous W, and 1.2 GPa for nanoporous CuZr [56]. Closer values of stress were observed for aluminum oxide nanoporous materials, where the oxide layer reaches stresses of ≈12 GPa [57], which is very close to a low sp${}^{3}$ or polymeric-like aC foam, such as that simulated in Figure 2. In addition, sp${}^{3}$ content dominates not only the stiffness, but also the transition from brittle to ductile failure. A significant difference was observed between the maximum tensile stress and the final stress at ϵ=0.5. Furthermore, the stress–strain curves show a rough fall in stress which becomes more prominent with the increase in sp${}^{3}$ bonding. These results can be attributed to the brittle nature of sp${}^{3}$ bonds, which cannot absorb plastic deformation, promoting embrittlement of the nanofoam. However, if we focus on the material toughness, it increases with sp${}^{3}$ content, suggesting that diamond-like materials could absorb more energy due to deformations, as expected from the differences between graphite and diamond. Despite the different elastic and plastic behavior, all curves converge to the same stress value for strains in the range of 0.45 to 0.50, indicating that plasticity under high-stress fields is independent of the sp${}^{3}$ content.

**Figure 3:**
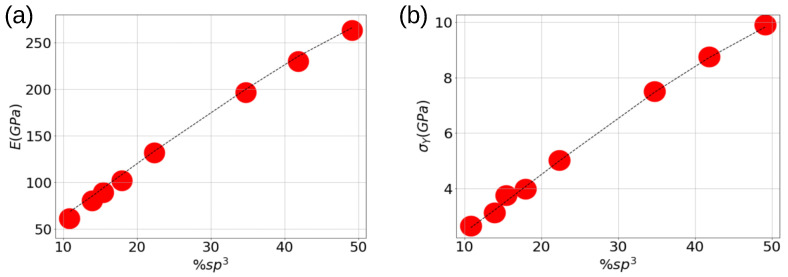
Comparison of MD results (red points) with theoretical models (lines) for Young’s modulus (**a**) and yield stress (**b**) during the foam fracture process respect to $\%sp^{3}$.

The elastic properties of carbon nanofoams are shown in Figure 3. As expected from the stress–strain curves, sp${}^{3}$ content determines the stiffness of the nanoporous material. Of course, the values of the elastic modulus are smaller than those observed in diamond and DLC. The literature exposes macroporous foams with weaker Young’s modulus under tension and compression compared with aC films [11,24]. A graphitic foam with mm size macropores and FTIR based $sp^{3}$ bonds gives an elastic modulus of 50 MPa [54]. A foam with macroporosity, with a size of 1 mm gives an elastic modulus of 2 MPa [55], all of them remarkably smaller than that observed here. As a cellular solid, we can invoke the Gibson–Ashby model to predict how porosity impacts the material Young’s modulus by means of the following:

(2) $$E=CE_{0}\left(\%{\mathrm{sp}}^{3}\right){\left(\rho /\rho_{0}\right)}^{3/2}\;,$$

where $E_{0}\left(\%{\mathrm{sp}}^{3}\right)$ is Young’s modulus of the bulk material for a given $sp^{3}$ and $\rho /\rho_{0}$ is the relative density of the nanoporous aC with respect to the bulk sample (no porosity). The term $E_{0}\left(\%{\mathrm{sp}}^{3}\right)$ depends on each simulated specimen, since in our simulations, sp${}^{3}$ is a variable parameter and $\rho /\rho_{0}$, the constant value (0.5), is determined by the porosity of the nanoporous material. He and Thorpe [58] proposed an analytical model to scale the elastic properties of covalent solids, where Young’s Modulus of a bulk ($E_{0}$) scales with the average network coordination (〈r〉) as

(3) $$E_{0}\left(\%{\mathrm{sp}}^{3}\right)=E_{0}{\left(\langle r\rangle -2.4\right)}^{\frac{3}{2}}$$

with the factor 2.4 corresponding to the transition from a rigid to a floppy network, and E${}_{0}$ Young’s modulus of the diamond. Considering that the average coordination (〈r〉) for amorphous carbon network scales with the sp${}^{3}$ fraction, $x_{{\mathrm{sp}}^{3}}$, as $\langle r\rangle =3+x_{{\mathrm{sp}}^{3}}$, the Young modulus in terms of the $x_{{\mathrm{sp}}^{3}}$ can be obtained by combining Equations (1) and (2) as

$$E=C_{E}E_{0}{\left(0.6+x_{{\mathrm{sp}}^{3}}\right)}^{3/2}{\left(\rho /\rho_{0}\right)}^{3/2}$$

The good agreement with the Young modulus determinations observed in Figure 3 indicates that the Gibson–Ashby model, initially proposed for macroscopic cellular solids, is also held for nanoscale amorphous carbon systems. Additionally, the nanofoams’ Young’s modulus scales with a composition obeying a $r^{3/2}$ power law as proposed by He and Thorpe [58]. The mixing of both models suggests that C networks could be successfully described by macroscopic models of porous materials. Additionally, we extended the same model to describe the yield strength as

$$\sigma_{Y}=C_{\sigma }\sigma_{0}{\left(0.6+x_{{\mathrm{sp}}^{3}}\right)}^{3/2}{\left(\rho /\rho_{0}\right)}^{3/2}$$

with $\sigma_{0}$ the yield stress of the bulk materials. It is straightforward to note the good agreement with the MD results shown in Figure 3b. The fitting parameters adopted for $E_{0}$ and $\sigma_{0}$, listed in Table 2, are slightly smaller than those reported for diamond materials, which is expected from the random network of the amorphous carbon structure. Table 2 also shows a very good percentage error in our fitting, below 4.5%. Furthermore, the constants $C_{0}$ agree with the reported values for nanoporous gold with open-pore and gyroidal-like nanostructure [49,59].

**Figure 4:**
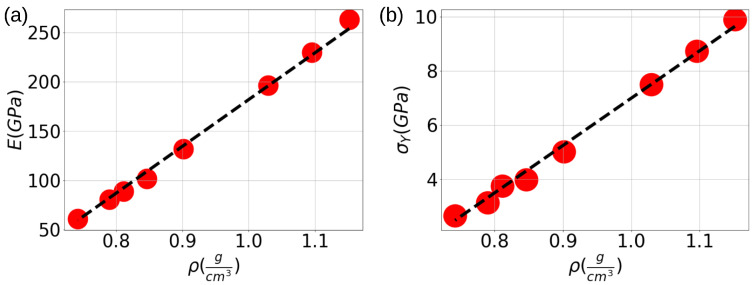
Comparison of (**a**). MD results (red points) with linear regression for Young Modulus (**a**) and Yield Stress (**b**) during the foam fracture process respecting density ρ.

Comparisons between MD and experiments are typically carried out by comparing amorphous carbon densities. Figure 4a,b summarizes the scaling laws in terms of the amorphous carbon density ρ. It is found that Young’s modulus scales almost linearly with ρ for all samples studied here using

(4) $$E\left(\rho \right)=a_{E}+b_{E}\rho$$

with $a_{E}=-293$ GPa and $b_{E}=475$ GPa·cm${}^{3}$/g. It is found that $\sigma_{Y}$ also follows a linear scaling law of the form

(5) $$\sigma_{Y}\left(\rho \right)=a_{\sigma }+b_{\sigma }\rho \;,$$

with $a_{\sigma }=-10.5$ GPa and $b_{\sigma }=17.5$ GPa·cm${}^{3}$/g. It is worth mentioning that (4) and (5) have similar nonphysical results for $\rho =a_{E}/b_{E}\leq 0$ or $\rho =a_{\sigma }/b_{\sigma }\leq 0$, leading in both cases to negative values of Young’s modulus and yield stress. The critical density for those results follows that $a_{E}/b_{E}\approx a_{\sigma }/b_{\sigma }\approx 0.6$ g/cm${}^{3}$. Such densities, in agreement with the He and Thorpe model [58], eliminate the elastic constants of covalent solids, rendering polymeric-like carbon instead of amorphous carbon with sp${}^{2}$ and sp${}^{3}$ bonding. Therefore, we expect that the scaling laws remain valid in the limit for graphitic or DLC-like carbon materials.

**Figure 5:**
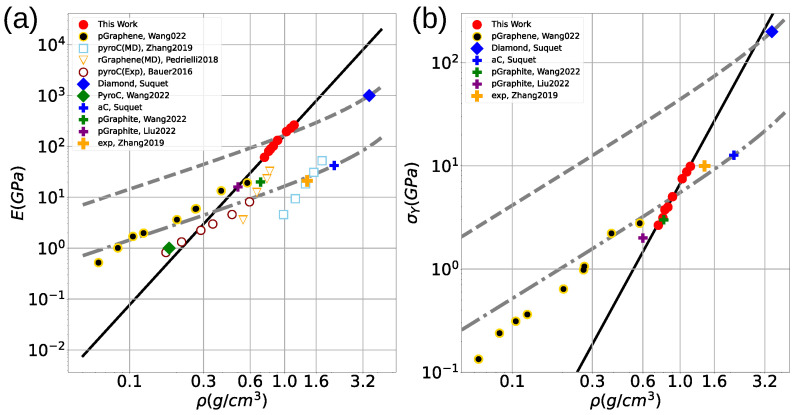
(**a**,**b**) show Young’s modulus and yield stress compared with other related works. Both upper and lower dashed lines correspond to the theoretical limit (Suquet equation) of diamond and graphite porous materials as a function of the density. The red points correspond to our simulation results. Points correspond to experiments and MD simulations provided by refs. [12,13,31,36,37,61].

The Suquet upper limit for yield stress ($\sigma_{y}^{SU}$) establishes a dependence with density on cellular solids that scales as the relative density as

(6) $$\sigma_{y}^{SU}=\frac{6\sigma_{y}\rho /\rho_{0}}{\sqrt{69-33\rho /\rho_{0}}}\;,$$

using $\sigma_{y}=200$ GPa and $\sigma_{y}=9$ GPa for diamond and graphite, respectively, Ref. [60], we establish the theoretical upper limits for graphite and diamond porous materials. As shown in Figure 5, nanoporous aC yield strength surpasses graphitic theoretical strength and becomes very close to the diamond upper limit for a cellular solid. Figure 5 shows that aC foams display a high elastic modulus, going beyond a simple Suquet scaling extrapolation for diamond foams. Regarding yield strength, our aC foams display remarkably high values. The high values for Young’s modulus are explained because our structures have filaments which include sp3 bonding, which controls deformation and ensures a much larger elastic modulus. Extrapolating to even lower densities, our results would appear roughly in line with the simulation results by Liu et al. [37] and Wang et al. [12], but here we cover higher densities and reach larger elastic modulus and yield values. There is a disagreement with low-density foams made from crystalline diamond blocks [37].

### Plastic Deformation

**Figure 6:**
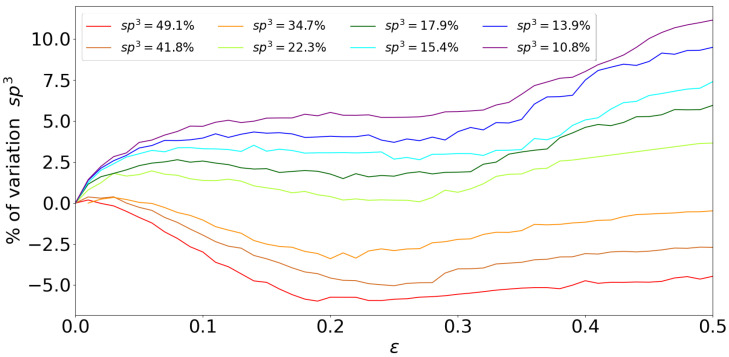
Variation of sp${}^{3}$ as a function of strain for the different nanoporous specimens.

In amorphous solids, plastic deformation cannot be directly defined. By inspecting the percentage variation in initial sp${}^{3}$ bonding, we found that all samples modify sp${}^{3}$ content as a function of strain (Figure 6). Furthermore, two groups can be directly identified. Samples with sp${}^{3}$ content smaller than 22% show diamond-like bonding that increases at small strains, then remains almost constant, and then increases again close to the nanofoam rupture point. The most relevant case occurs for smaller sp3 content, where samples are mostly sp${}^{1}$ and sp${}^{2}$. Since our samples are hydrogen-free, a large stress field can turn the bonding recombination to form sp${}^{3}$ from C atoms with sp and sp${}^{2}$ coordination. Since Figure 6 shows the percentage variation of sp${}^{3}$, we note that the total increase of sp${}^{3}$ relative to the total number of bonds present in each sample is not higher than 1–2% for strains close to 0.5. Samples with sp${}^{3}$ higher than 34.7% show an opposite behavior since deformation promotes the destruction of diamond bonds as a strain function.

**Figure 7:**
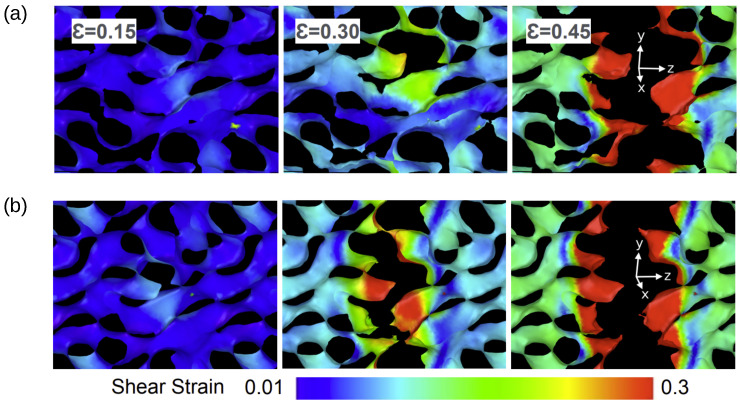
Shear strains for samples with (**a**) sp${}^{3}=$ 22.3% and (**b**) sp${}^{3}$ = 49.1% Snapshots correspond to strains of 0.15, 0.30, and 0.45. Color coding represents the atomic shear strain.

As commented from the stress–strain curves, low sp${}^{3}$ samples present a delayed fracture compared to high sp${}^{3}$ samples. In Figure 7, it is shown that for both cases (22.3 and 49.1% sp${}^{3}$) there are no major differences at strains closer to 0.15. However, as the stress increases, differences in fracturing become notorious. At ϵ=0.30, sp${}^{3}$ = 49.1% shows the failure of several filaments in a zone characterized by a high concentration of shear strain. For sp${}^{3}$ = 22.3, shear strain is weaker and filaments are still connected. For 0.45, is observed that failure is independent of sp${}^{3}$, revealing a brittle crack without evidence of filament necking.

**Figure 8:**
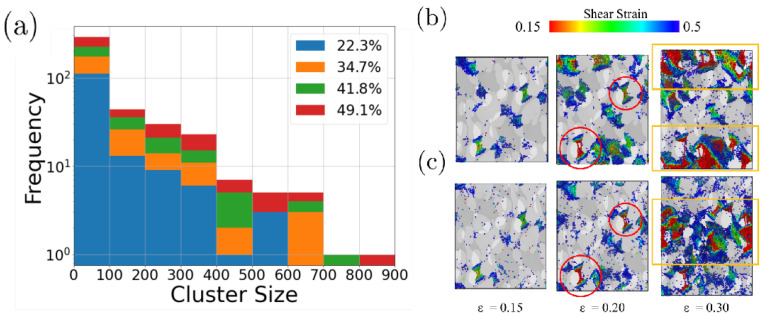
(**a**) Histograms showing the number of clusters of atoms with shear strain larger than 0.15. The shear strain of a half-cut of the nanoporous aC with (**b**) $sp^{3}$ = 22.3% and (**c**) $sp^{3}$ = 49.1% at different strains. Atoms are colored according to shear strain. Atoms with shear lower than 0.15 were removed from the figure and not considered in the cluster count. Red circles illustrate the breaking of some filaments at strains of 0.15. Orange regions show the formation of cracks at strains of 0.30.

To unveil the differences in the failure of the carbon nanofoams, we inspected the role displayed by atoms with large accumulations of shear strain. To this end, atoms with shear strength lower than 0.15 were removed and the remaining atoms were grouped into clusters when the distance to their neighbors was smaller than 2.0 Å. Notably, samples with higher sp${}^{3}$ tend to present larger clusters compared to samples with lower sp${}^{3}$. This suggests that diamond-like structures tend to induce shear localization at earlier stages of deformation. Additionally, smaller clusters are also likely to appear in high sp${}^{3}$ samples. A visual inspection of the shear strain is provided in Figure 8b,c for the $sp^{3}$ = 22.3% and $sp^{3}$ = 49.1% samples, respectively, where numerous single atoms with high shear strength are distinguished. When separating the contribution of single atoms in the cluster count of sizes <100, it follows that low sp${}^{3}$ samples present a higher number of sheared atoms homogeneously distributed across the sample. This is similar to the observations of nanoindentation of DLC surfaces [24].

At strains of 0.2, the presence of larger high shear strain clusters is observed and the precursors of the nanofoam failure can be clearly identified. The red circles show the same shear clusters corresponding to the break of filaments assisted by carbon shear for samples with sp${}^{3}$ = 22.3 and 44.9%. Almost every high shear cluster appears in the same region independent of the sp${}^{3}$ composition, which means that at small strains, plastic deformation is dominated by geometry more than by sp${}^{3}$ composition. This scenario changes at larger strains, where the shear distribution differs for both samples (orange boxes). At higher strains, shear is highly localized in the sp${}^{3}=49.1\%$ sample, whereas the large number of isolated shear atoms in the sp${}^{3}\;22.3\%$ specimens leads to the nucleation of larger clusters in different parts of the nanofoams.

**Figure 9:**
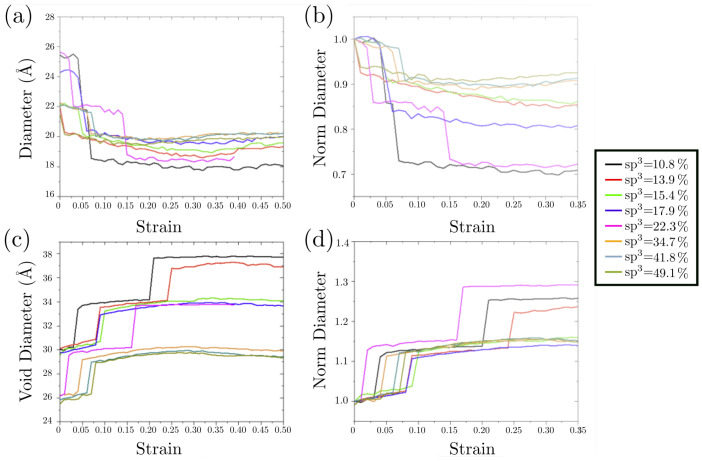
Ligament and (**a**,**c**) void diameters and the corresponding normalized diameters (**b**,**d**) for the simulated nanofoams.

To study the role displayed by the nanofoam topology, we conducted an exhaustive study of voids and filaments as functions of strain with the FoamExplorer code, as shown in Figure 9. Figure 9a shows that, at early stages of deformation, the stress–strain curve is not only dominated by smaller changes in coordination but also by the decrease in the mean diameter of the foam filaments and the increase in the void size Figure 9b. The abrupt fall in the curves is attributed to the average of multiple filaments either breaking or reducing their diameter. The analysis of the normalized void and filament diameter shows that filaments can decrease at least 30% with respect to their initial size, which is consistent with the increase in the void in Figure 9d. Additionally, the mean void size reveals that higher sp${}^{3}$ samples present smaller variations in the void size due to the lower ductility of diamond-like materials in comparison with the graphitic ones.

**Figure 10:**
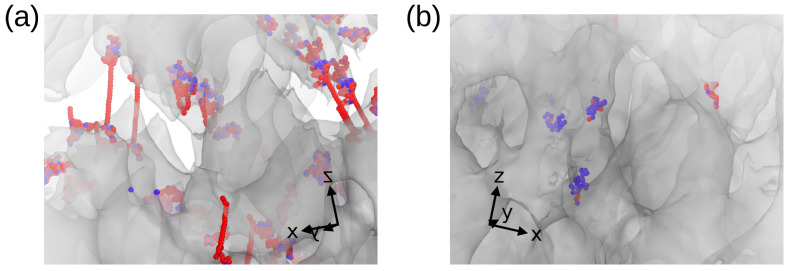
Bond breaking during the fracture process of the filaments. Snapshots correspond to the strain of 0.35 for samples of 49.1% (**a**,**b**) of 10.8% of $sp^{3}$. Around the fracture, atoms are colored according to their coordination, which is lower than other atoms in their neighborhood. Red and blue atoms show coordination 1, and 2, respectively.

To unveil the atomistic phenomena involved in filament breaking, visual inspection was carried out as shown in Figure 10 for samples with sp${}^{3}$ content of 10.8% and 49.1%. From Figure 10a, the atoms responsible for filament breaking on a sample of sp${}^{3}$ = 44.9% are illustrated in blue and red, and correspond to groups of atoms with coordination of 2 and 1, respectively. Based on these observations, previous to the system failure there was a group of atoms per filament with low coordination (blue and red atoms), which are mainly responsible for filament breaking. On the other hand, the sample with sp${}^{3}$ = 10.8% shows a larger number of sp${}^{1}$ atoms (carbon chains) during filament breaking (Figure 10b). It is worth mentioning that those carbon chains originated on regions of high shear strain localization, and turn, on the enhanced ductility of low sp${}^{3}$ samples compared with the diamond-like nanofoams observed previously.

## 4. Conclusions

Atomistic simulations were conducted to model amorphous carbon nanofoams with sp${}^{3}$ contents of 10–50%. The results reveal that sp${}^{3}$ directly controls the mechanical response for elastic modulus, ultimate tensile stress, and ductility. Interestingly, the relatively smooth Lorentzian-shape curves for the stress–strain of graphene networks [36,37] are different from the curves for the aC foams with high sp${}^{3}$ content simulated here, where there is a sharp decrease in stress, indicating a fracture due to the bond breaking of the diamond-like bonding.

The simulated nanofoams reveal an ultimate tensile stress in the range of 10–20 GPa modulated by the sp${}^{3}$, which is, as far as we know, higher than other nanoporous structures with porosities close to those reported here (50%). The ultra-high strength is due to the diamond-like structure of the filaments, which make their mechanical properties scale with the carbon coordination. Our findings show that amorphous carbon nanofoam’s Young’s modulus and yield stress can be quantitatively described in terms of the Gibson–Ashby model for cellular solids together with the He and Thorpe [58] equation for covalent materials.

Atomistic simulations also reveal that the failure mechanism is strongly dependent on the sp${}^{3}$ content. Low sp${}^{3}$ samples show homogeneous shear localization, forming high shear stress clusters with a relatively small number of carbon atoms. On the other hand, high-sp${}^{3}$ nanofoams are prone to produce larger shear strain clusters in localized regions, which is consistent with the brittle fracture observed from the stress–strain curves. Shear strain accumulation, which grows with strain, produces bond breaking inside the filaments to finally drive the filament fractures. All in all, our simulations show that amorphous carbon nanofoams rise as mechanical materials with exceptional mechanical properties which can be controlled in terms of porosity and sp composition, leading to materials with ultra-high strength in terms of their relatively low density.

## Figures and Tables

**Table 1:** Calculated density of carbon foams with a porosity of 50% for each $\%sp^{3}$ content.

$\%{\mathrm{sp}}^{3}$	$\rho (\frac{g}{{\mathrm{cm}}^{3}})$
10.8	0.742
13.9	0.790
15.4	0.811
17.9	0.846
22.3	0.901
34.7	1.029
41.8	1.095
49.1	1.152

**Table 2:** Elastic constants and fitting parameters with the corresponding errors.

Elastic Constant	Fitting Parameters	Δ(%)
*E*	$E_{0}=757$ GPa $C_{0}=0.94$	4.42
$\sigma_{Y}$	$\sigma_{0}=88$ GPa $C_{0}=0.30$	1.93

## Data Availability

Data will be available by request to the corresponding author.

## Supplementary Materials

The following supporting information can be downloaded at https://www.mdpi.com/article/10.3390/nano13081429/s1, Figure S1: Von Mises strain maps for different sp${}^{3}$ nanofoams.
