# Supplementary material for: Nanoporous Amorphous Carbon with Exceptional Ultra-High Strength
Source: Nanomaterials (Basel). 2023 Apr 21;13(8):1429. doi: 10.3390/nano13081429 (PMC10142945; doi:10.3390/nano13081429)
Supplement: Supplementary file 1 [file nanomaterials-13-01429-s001.zip › nanomaterials-2316005-supplementary.pdf]

# Supplementary Material

## Nanoporous amorphous carbon with exceptional ultra-high strength

Daniel Castillo-Castro, Felipe Correa, Emiliano Aparicio, Nicolas Amigo, Alejandro Prada, Rafael I. Gonzalez, Eduardo Bringa, and Felipe J. Valencia

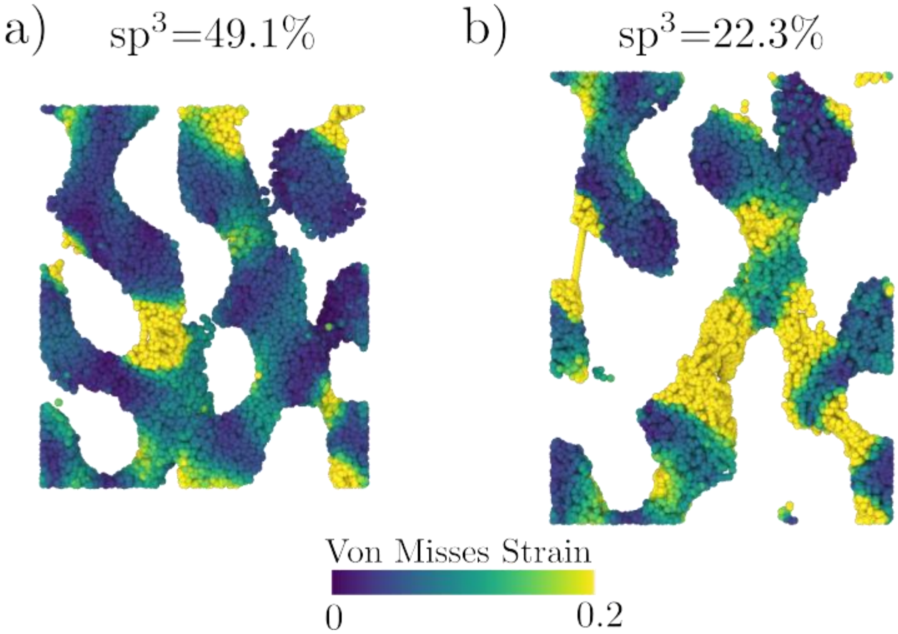


Figure S1. Von Mises strain for a nanoporous aC with $sp^3$ of 49.1% (a) and 22.3% (b). In figure, a) represent a strain of 0.2, while b) a strain of 0.28.
